# Relationship between spontaneous sympathetic baroreflex sensitivity and cardiac baroreflex sensitivity in healthy young individuals

**DOI:** 10.14814/phy2.12536

**Published:** 2015-11-12

**Authors:** Chloe E Taylor, Trevor Witter, Khadigeh El Sayed, Sarah L Hissen, Aaron W Johnson, Vaughan G Macefield

**Affiliations:** 1 School of Science and Health, University of Western SydneySydney, Australia; 2 Centre for Translational Physiology, University of OtagoWellington, New Zealand; 3 School of Medicine, University of Western SydneySydney, Australia; 4 Neuroscience Research AustraliaSydney, Australia

**Keywords:** Blood pressure, heart rate, muscle sympathetic nerve activity, sequence method

## Abstract

Low baroreflex sensitivity (BRS) is associated with elevated cardiovascular risk. However, the evidence is based primarily on measurements of cardiac BRS. It cannot be assumed that cardiac or sympathetic BRS alone represent a true reflection of baroreflex control of blood pressure. The aim of this study was to examine the relationship between spontaneous sympathetic and cardiac BRS in healthy, young individuals. Continuous measurements of blood pressure, heart rate, and muscle sympathetic nerve activity (MSNA) were made under resting conditions in 50 healthy individuals (18–28 years). Sympathetic BRS was quantified by plotting MSNA burst incidence against diastolic pressure (sympathetic BRS_inc_), and by plotting total MSNA against diastolic pressure (sympathetic BRS_total_). Cardiac BRS was quantified by plotting R-R interval against systolic pressure using the sequence method. Significant sympathetic BRS_inc_ and cardiac BRS slopes were obtained for 42 participants. A significant positive correlation was found between sympathetic BRS_inc_ and cardiac BRS (*r *=* *0.31, *P *=* *0.049). Among this group, significant sympathetic baroreflex slopes were obtained for 39 participants when plotting total MSNA against diastolic pressure. In this subset, a significant positive correlation was observed between sympathetic BRS_total_ and cardiac BRS (*r *=* *0.40, *P *=* *0.012). When males and females were assessed separately, these modest relationships only remained significant in females. Analysis by gender revealed correlations in the females between sympathetic BRS_inc_ and cardiac BRS (*r *=* *0.49, *P *= 0.062), and between sympathetic BRS_total_ and cardiac BRS (*r *=* *0.57, *P *=* *0.025). These findings suggest that gender interactions exist in baroreflex control of blood pressure, and that cardiac BRS is not appropriate for estimating overall baroreflex function in healthy, young populations. This relationship warrants investigation in aging and clinical populations.

## Introduction

The baroreflex acts to regulate blood pressure, primarily through the modulation of heart rate and sympathetic outflow to the vasculature. The two arms of the baroreflex, cardiac and sympathetic, share the same afferent pathway, in which baroreceptors in the carotid sinuses and aortic arch detect pressure-driven increases in radial distension. Baroreceptor afferents project via the glossopharyngeal and vagus nerves to the nucleus tractus solitarius (NTS) within the medulla, from which excitatory projections synapse within the caudal ventrolateral medulla (CVLM), nucleus ambiguous (NA), and the dorsal motor nucleus of the vagus (DMX) (Andresen and Kunze 1994). The excitatory sign of the baroreceptor afferents is reversed at the level of the rostral ventrolateral medulla (RVLM), the primary output nucleus for muscle sympathetic nerve activity (MSNA) (Dampney et al. 2003; Macefield and Henderson 2010), to which inhibitory projections from the CVLM project and lead to withdrawal of sympathetic outflow to the muscle vascular bed. Reversal of the sign of the baroreceptor afferent input also occurs at the level of the sinoatrial node, via the release of the acetylcholine from terminals of the cardiac vagus nerve (Blessing 2003). As such, the baroreflex plays a critically important role in regulating blood pressure constant, though its sensitivity can be adjusted to suit current physiological needs. The sensitivity of this negative feedback loop system can be assessed by quantifying the relationship between systolic blood pressure and heart rate (or R-R interval) (Smyth et al. 1969; Hunt et al. 2001a), and between diastolic blood pressure and MSNA (Kienbaum et al. 2001; Studinger et al. 2009).

Low baroreflex sensitivity (BRS) is associated with elevated cardiovascular risk (Bristow et al. 1969; Eckberg et al. 1971; Lantelme et al. 2002; Johansson et al. 2007; La Rovere et al. 2008). However, the evidence is based primarily on measurements of cardiac BRS alone. It cannot be assumed that cardiac or sympathetic BRS on their own represent a true reflection of baroreflex control of blood pressure. Aging has been associated with a fall in cardiac BRS due to arterial stiffness and therefore a reduced capacity for the baroreceptors to encode changes in arterial pressure (Monahan et al. 2001). In theory, arterial stiffness with aging ought to affect sympathetic BRS for the same reasons. However, Studinger et al. (2009) reported that sympathetic BRS is maintained in older individuals due to a compensatory increase in the “neural component” of the baroreflex response. While it is not clear in which part of the neural response the increase can be attributed to (afferent, central, and/or efferent), the finding does suggest that poor baroreflex control of heart rate does not necessarily imply poor baroreflex control of MSNA.

To the best of our knowledge, there have been only two studies to date in which cardiac and sympathetic BRS have been directly compared. Both Rudas et al. (1999) and Dutoit et al. (2010) reported that there is no correlation between the two in the healthy individuals. The authors of these two studies employed the modified Oxford method for the assessment of cardiac and sympathetic BRS. Although Rudas et al. (1999) also included spontaneous methods of assessing BRS, significant sympathetic baroreflex slopes were reported in only five of the 18 participants, thus limiting the capacity to examine the relationship with spontaneous cardiac BRS. The modified Oxford method is a pharmacological technique, which involves administering bolus injections of sodium nitroprusside and phenylephrine in order to cause blood pressure to fall and subsequently rise. The beat-to-beat heart rate and MSNA responses to this active perturbation of blood pressure allow BRS to be quantified. The modified Oxford method is typically referred to as the gold standard approach for the assessment of cardiac BRS because it allows baroreflex responses to be quantified during rapid changes in arterial pressure (Diaz and Taylor 2006). However, this approach can have limitations when applied to sympathetic BRS testing because of fewer data points available to produce a baroreflex slope, particularly in response to rising pressures. In contrast to the assessment of cardiac BRS, where each cardiac cycle is associated with an R-R interval, the assessment of sympathetic BRS relies upon the occurrence of bursts of MSNA, which do not occur with every cardiac cycle and which vary in their incidence across individuals. This issue is particularly troublesome at higher pressures when there is significant inhibition of sympathetic bursts (Dutoit et al. 2010). The alternative is to use spontaneous techniques, and these are frequently used for the assessment of sympathetic BRS (Kienbaum et al. 2001; Keller et al. 2006; Hart et al. 2010, 2011b). Spontaneous fluctuations in diastolic pressure and MSNA at rest are used to quantify sympathetic BRS using a significantly larger number of cardiac cycles, to construct the baroreflex slope. When examining the relationship between cardiac and sympathetic BRS, it is logical that the same type of approach be used to assess the two arms of the baroreflex. To the best of our knowledge, there has yet to be a study in which this relationship has been investigated with the use of spontaneous baroreflex techniques.

In the current study, we have used two methods to assess spontaneous sympathetic BRS: one approach involves the use of MSNA burst incidence and the other total MSNA. Previous research indicates that plotting MSNA burst incidence against diastolic pressure results in a greater number of significant baroreflex slopes, compared with plotting MSNA burst amplitude or area against diastolic pressure (Kienbaum et al. 2001). However, using total MSNA allows both MSNA burst amplitude and MSNA burst incidence to be taken into account in the assessment of BRS (Keller et al. 2006). Therefore, we will use the total MSNA method as well as the more common MSNA burst incidence method to examine the relationship with cardiac BRS. It is hypothesized that spontaneous sympathetic BRS is correlated with spontaneous cardiac BRS in healthy, young individuals.

## Methods

### Participants

Fifty healthy young males (*n *=* *31) and females (*n *=* *19) aged 18–28 years were recruited for the study. Exclusion criteria included diagnosed cardiovascular, respiratory, or endocrine disease and those who smoked or took regular medication. Participants were instructed to abstain from alcohol or vigorous exercise 24 h prior and to not consume any caffeine on the day of the experiment. All experiments took place in the morning, beginning between 0800 and 0900 h, as we have previously demonstrated that diurnal variation exists in cardiac BRS (Taylor et al. 2011). The changes in hormone levels during the menstrual cycle have been shown to affect MSNA and sympathetic BRS (Minson et al. 2000b). Accordingly, females were tested in the low hormone (early follicular) phase of their menstrual cycle to minimize the effects of sex hormones on BRS. Written informed consent was obtained from all participants prior to conducting the experiment, who were reminded that they could withdraw at any time. The study was conducted with the approval of the Human Research Ethics committee, University of Western Sydney, and satisfied the Declaration of Helsinki.

### Measurements and experimental protocol

**Figure 1:**
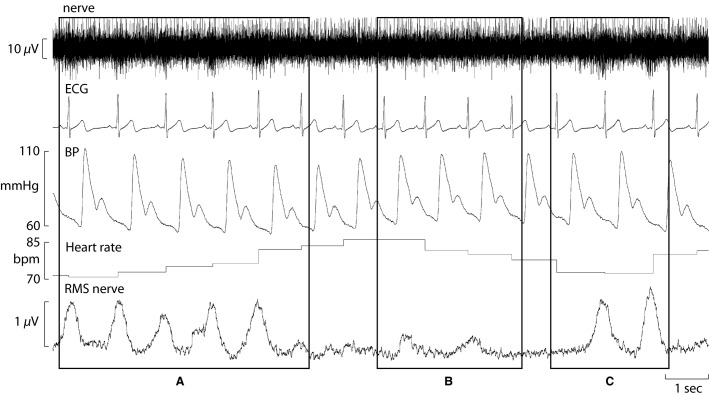
Experimental records from a 22-year-old male at rest. The nerve signal has been shifted by 1.2 sec to account for sympathetic baroreflex conduction delay. The baroreflex drives a shortening of R-R intervals (increase in heart rate) and increase in MSNA burst incidence in response to falling systolic and diastolic pressures (A). A lengthening of R-R intervals (decrease in heart rate) and inhibition of MSNA bursts occurs in response to rising systolic and diastolic pressures (B). MSNA burst incidence increases in response to falling diastolic pressures despite maintained systolic pressure (C), demonstrating that MSNA is mostly strongly related to diastolic pressure.

Participants were studied in an upright-seated position in a comfortable chair, with the legs supported in the extended position. Continuous MSNA recordings were made from muscle fascicles of the common peroneal nerve through tungsten microelectrodes (FHC, Bowdoinham, ME, USA) inserted percutaneously at the level of the fibular head. Multiunit neural activity was amplified (gain 20 000, bandpass 0.3–5.0 kHz) using an isolated amplifier (Neuroamp EX, ADInstruments, Sydney, Australia) and stored on computer (10 kHz sampling rate) using a computer-based data acquisition and analysis system (Powerlab 16SP hardware and LabChart 7 software; ADInstruments, Sydney, Australia). A root mean square (RMS) processed version of this signal was computed, with a moving average of 200 msec. Blood pressure was recorded noninvasively via a finger cuff (Finometer; Finapres Medical System, Amsterdam, the Netherlands). Heart rate was recorded via electrocardiogram (0.3–1.0 kHz, Ag-AgCl surface electrodes, sampled at 2 kHz). Respiration was measured via a strain-gauge transducer (Pneumotrace, UFI, Morro Bay, CA) wrapped around the chest. A minimum of 10 min of resting data were recorded in order to examine spontaneous fluctuations in blood pressure and the corresponding changes in R-R interval and MSNA (Fig.1). Participants were asked to breathe normally throughout.

### Data analysis

Beat-to-beat values were extracted from LabChart (ADInstruments, Sydney, Australia) for systolic blood pressure, diastolic blood pressure, R-R interval, and MSNA. A custom-written program, developed in LabView software (National Instruments), was used to detect and measure the area of individual bursts of MSNA. The numbers of bursts per minute (MSNA burst frequency) and per 100 heartbeats (MSNA burst incidence) were determined for each individual. Total integrated MSNA was determined using a segregated signal averaging approach described by Halliwill (2000), whereby the largest MSNA burst during the 10-min rest period was assigned a value of 1000 and a prolonged section without bursts was assigned a value of zero. The remaining MSNA bursts were calibrated against this to allow measures of MSNA to be normalized to individual resting values. The measurement of total MSNA allows both MSNA burst incidence and MSNA burst amplitude to be taken into account when quantifying MSNA for a given diastolic pressure bin.

### Sympathetic baroreflex sensitivity: burst incidence method

Sympathetic BRS was quantified using methods previously described by Kienbaum et al. (2001);. For all methods of assessing sympathetic BRS, the nerve trace was shifted to account for the sympathetic baroreflex conduction delay, and this was adjusted for each participant to account for interindividual differences in burst latency. The average shift applied was 1.28 ± 0.01 sec, relative to the R-wave to which the sympathetic burst was aligned. For each participant, the diastolic pressure values for each cardiac cycle throughout the 10-min rest period were assigned to 3 mmHg bins to reduce the influence of respiratory-related oscillations (Ebert and Cowley 1992; Tzeng et al. 2009). For each bin the corresponding MSNA burst incidence (number of bursts per 100 cardiac cycles) was determined. Sympathetic BRS was quantified by plotting MSNA burst incidence against the mean diastolic blood pressure for each bin. Each data point was weighted according to the number of cardiac cycles because the bins at the highest and lowest diastolic pressures contain fewer cardiac cycles (Kienbaum et al. 2001). Baroreflex slopes were determined using linear regression with the acceptance level set at *r *>* *0.5 (Hart et al. 2011b). The value of the slope provided the sympathetic BRS for the individual, which will be referred to as “sympathetic BRS_inc_” in order to differentiate from other methods of determining sympathetic BRS.

### Sympathetic baroreflex sensitivity: total MSNA method

**Figure 2:**
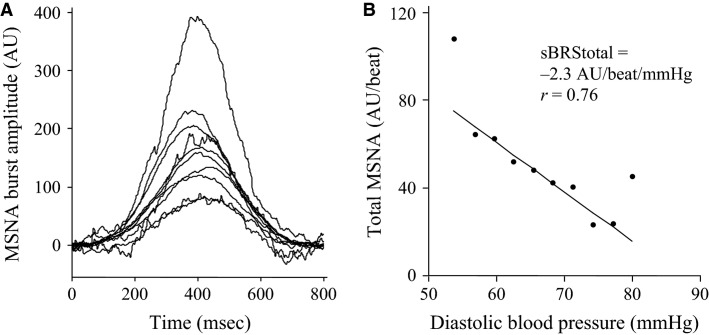
Sympathetic baroreflex assessment in a 21-year-old male using the segregated signal averaging approach.MSNA bursts are normalized to the burst with the largest amplitude and entered into diastolic pressure bins of 3 mmHg (A). Total MSNA per beat is determined for each bin and plotted against diastolic pressure (B).

The relationship between diastolic blood pressure and total MSNA was assessed using 3 mmHg bins. Since all cardiac cycles are incorporated in this analysis, including those not associated with MSNA bursts, this measure of total MSNA takes into account both MSNA burst incidence and MSNA burst amplitude. Figure2A illustrates the mean MSNA burst amplitudes for each diastolic pressure bin for one individual. The lowest diastolic pressure bins are associated with the largest MSNA bursts, with the average burst amplitude becoming progressively smaller with high diastolic pressures. Total integrated MSNA was determined for each bin using segregated signal averaging approach (Halliwill 2000) and expressed as arbitrary units (AU) per beat. Linear regression was used to determine the relationship between total MSNA and diastolic blood pressure with the application of the weighting procedure described above and an acceptance level of *r *>* *0.5. Figure2B illustrates the baroreflex slope for one individual. These baroreflex values will be referred to as “sympathetic BRS_total_” in order to differentiate from the MSNA burst incidence method for assessing sympathetic BRS.

### Cardiac baroreflex sensitivity: sequence method

Cardiac BRS was assessed using the sequence method in which “up” and “down” sequences are identified. Up sequences consist of three or more consecutive cardiac cycles for which there is a sequential rise in both systolic pressure and R-R interval. Down sequences consist of three of more cardiac cycles for which there is a sequential fall in systolic pressure and R-R interval. The threshold for changes in systolic BP was set at 1 mmHg and the threshold for changes in R-R interval was set at 6 msec (Parati et al. 1988). Sequences containing changes smaller than these thresholds were not used in the assessment of cardiac BRS. Baroreflex sensitivity was quantified by plotting R-R interval against systolic pressure for each sequence (*r *≥* *0.8 acceptance level) and taking the average slope value for up and down sequences combined. Values of cardiac BRS were accepted when the number of sequences was ≥3 for both up and down sequences.

### Statistical analysis

Linear regression analysis was used to examine the relationships between sympathetic BRS and cardiac BRS. Subgroup analyses were performed to assess these relationships for males and females separately. All statistical analyses were performed using SPSS v22. For all statistical tests, a probability level of *P *<* *0.05 was regarded as significant. Values are presented as mean ± SE.

## Results

### Participants

Recordings of MSNA were successfully obtained in all 50 participants. Sympathetic baroreflex slopes (*r *>* *0.5) were successfully obtained for 48 participants using the burst incidence method. For six participants, the number of cardiac BRS sequences was <3 for up and/or down sequences, and thus data for these participants were removed from the analysis, leaving a total of 42 (27 males). For these participants, the mean number of cardiac BRS sequences was 29 ± 3. Resting cardiovascular and sympathetic variables for these 42 participants are presented in Table1. There were no significant differences between males and females except for resting MSNA, which was significantly higher in males when expressed as both MSNA burst frequency and MSNA burst incidence (*P *<* *0.01). Mean body mass index (BMI) was above 25 kg/m^2^, and thus in the overweight category. However, fat-free mass was 67.4 ± 9.1 kg for males and 49.8 ± 5.8 kg for females. These values are above average for healthy, young individuals (Kyle et al. 2001), which can be explained by the physical activity levels of the sample. Subjects exercised regularly (≥2 ×  per week), with several partaking in resistance exercise. Spontaneous cardiac BRS and sympathetic BRS values are presented in Table2. There were no significant differences between males and females (*P *>* *0.05).

**Table 1:** Resting cardiovascular and sympathetic variables

Variable	All participants (n = 42)	Males (n = 27)	Females (n = 15)	P
Age (years)	22 ± 0.5	22 ± 0.4	23 ± 0.9	0.30
BMI (kg/m^2^)	25.6 ± 0.8	25.1 ± 0.6	26.6 ± 2.0	0.48
Systolic BP (mmHg)	121 ± 4	121 ± 4	121 ± 7	1.0
Diastolic BP (mmHg)	76 ± 2	76 ± 3	77 ± 4	0.74
Heart rate (beats/min)	69 ± 1	67 ± 2	71 ± 3	0.13
MSNA burst frequency (bursts/min)	37 ± 2	40 ± 2	33 ± 2	0.009*
MSNA burst incidence (bursts/100 heart beats)	55 ± 2	60 ± 2	45 ± 4	0.002*

BMI, body mass index; BP, blood pressure; MSNA, muscle sympathetic nerve activity

* Significant difference between males and females (*P* < 0.05).

**Table 2:** Cardiac and sympathetic baroreflex sensitivities

Baroreflex sensitivity	All participants (n = 42)	Males (n = 27)	Females (n = 15)	P
Cardiac BRS (msec/mmHg)	14.6 ± 0.9	14.0 ± 1.0	15.7 ± 1.7	0.33
Sympathetic BRS_inc_ (bursts/100 heart beats/mmHg)	−1.94 ± 0.21	−1.70 ± 0.24	−2.38 ± 0.38	0.12
Sympathetic BRS_total_(AU/beat/mmHg)	−2.45 ± 0.22*	−2.32 ± 0.26*	−2.65 ± 0.39	0.47

BRS, baroreflex sensitivity; AU, arbitrary units.

* Sample size is 39 (all) and 24 (males).

### Relationship between sympathetic and cardiac baroreflex sensitivity

**Figure 3:**
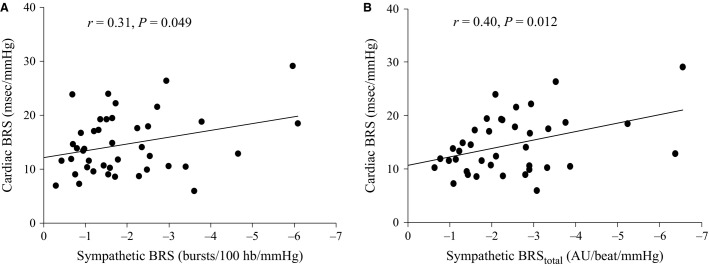
Relationship between cardiac and sympathetic baroreflex sensitivities (BRS) when using the MSNA burst incidence method (A) and the total MSNA method (B) for assessing sympathetic BRS for all male and female participants.

A significant positive correlation was found between cardiac BRS and sympathetic BRS_inc_ (*r *=* *0.31, *P *=* *0.049; Fig.3A). In 39 participants (24 males) significant sympathetic baroreflex slopes were obtained when using the total MSNA method. In this subset, correlations were observed between sympathetic BRS_total_ and cardiac BRS (*r *=* *0.40, *P *=* *0.012; Fig.3B).

### Gender interactions

**Figure 4:**
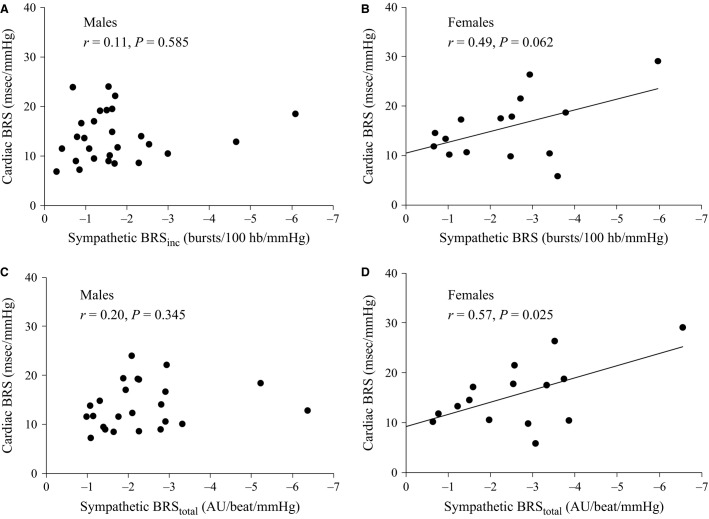
Relationship between cardiac and sympathetic baroreflex sensitivities (BRS) in males (A, C) and females (B, D) when using the MSNA burst incidence method and the total MSNA method for assessing sympathetic BRS.

When the relationship between cardiac BRS and sympathetic BRS_inc_ was assessed separately for males and females, there was no significant correlation for males (*r *=* *0.11, *P *=* *0.585; Fig.4A). Conversely, for females there was a positive relationship between cardiac BRS and sympathetic BRS_inc_ (*r *=* *0.49; Fig.4B), although this failed to reach statistical significance (*P *=* *0.062). In the subset of 39 participants who exhibited significant sympathetic baroreflex slopes, calculated from total MSNA, there was a significant correlation between sympathetic BRS_total_ and cardiac BRS for females (*r *=* *0.57, *P *=* *0.025; Fig.2D) but not males (*r *=* *0.20, *P *=* *0.345; Fig.2C).

## Discussion

We have examined, for the first time, the relationship between spontaneous cardiac and sympathetic baroreflex sensitivity. While the initial results indicate a relationship between cardiac and sympathetic BRS in young individuals, when assessed according to gender this modest relationship is evident only in females. Assessment of sympathetic BRS using both MSNA burst incidence and total MSNA yielded similar results. The findings suggest that cardiac BRS may only predict a small portion of the variance in sympathetic BRS in this group. This study indicates that gender interactions exist in baroreflex control of blood pressure, and that cardiac BRS is not appropriate for estimating overall baroreflex function in healthy, young populations.

### Relationship between cardiac and sympathetic baroreflex sensitivities

The cardiac and sympathetic baroreflexes share the same afferent pathway. It therefore seems logical that an individual would be effective in regulating blood pressure with both arms of the baroreflex, or be less effective with both. The current study findings suggest that in young females there is a relationship between cardiac and sympathetic BRS, and this could be attributed to the common afferent pathway. However, a considerable portion of the variance in BRS remains unexplained, and thus it appears there are other factors influencing the central integration of the baroreceptor input and the efferent pathways that lead to differences in cardiac and sympathetic BRS within individuals. It is also unclear as to why no relationship appears to exist between the two arms of the baroreflex in young males. The ability to regulate BP through the modulation of heart rate and MSNA appear to be quite separate, and the hypothesis that high cardiac BRS is indicative of high sympathetic BRS is therefore rejected.

### Gender interactions

Dutoit et al. (2010) reported no direct relationship between cardiac and sympathetic BRS in young individuals when both genders were investigated as one group. It is possible that the methods used may explain the discrepancy with the current findings; in the study by Dutoit et al. (2010) participants lay in the supine position, whereas in the current study participants were in an upright seated position. We have previously shown that posture significantly affects cardiac BRS (Taylor et al. 2013). Furthermore, resting MSNA is lower in the supine position (Ray et al. 1993), which may reduce the number of MSNA bursts with which to produce a sympathetic baroreflex slope. Despite this, in both studies, gender-based differences were apparent when separate analyses were performed for males and females. Consistent with the findings of Dutoit et al. (2010), the current study indicates that there is a positive relationship between cardiac BRS and sympathetic BRS in young females; a relationship that was not found in young males in either study.

There is evidence to suggest that cardiovascular control, particular at the level of the vasculature, differs between young males and females. Hart et al. (2009) reported that MSNA is correlated with total peripheral resistance in males but not females. Later, the same group demonstrated that *β*-adrenergic vasodilation blunts sympathetic vasoconstriction in young females (Hart et al. 2011a), thus providing an explanation for the lack of correlation between MSNA and total peripheral resistance. The authors reported that this mechanism was not apparent in young men or postmenopausal women. This infers that in young females with a high sympathetic BRS, baroreflex control of blood pressure via MSNA may not necessarily be more effective. An increase in sympathetic outflow to the vasculature is more likely to be counteracted by local vasodilator mechanisms than it would in their male counterparts.

Vascular transduction is a key step in the baroreflex response that is not taken into account with conventional methods of assessing sympathetic BRS. The inclusion of ultrasound measurements of vessel diameter and blood flow to determine the direct effects of MSNA on peripheral resistance (i.e., end-organ responsiveness), may help to explain gender-based differences in the relationship between cardiac and sympathetic BRS. In the current study, young males had significantly higher levels of resting MSNA than young females, as has been reported previously (Hogarth et al. 2007). This may highlight the reliance on local vasodilator mechanisms in females for adjusting vascular tone under resting conditions.

While the mechanisms surrounding gender differences remain somewhat speculative, the current findings suggest that potential gender interactions ought not to be ignored when investigating blood pressure regulation.

### Methodological considerations

The purpose of the current study was to use spontaneous techniques to assess the relationship between cardiac and sympathetic BRS. Spontaneous techniques specifically target the regulation of blood pressure under normal resting conditions. In contrast, the modified Oxford method involves bolus injections of sodium nitroprusside followed 60 sec later by phenylephrine, generally producing a fall in arterial pressure of ∼15 mmHg and a subsequent rise of ∼15 mmHg above baseline. The modified Oxford method therefore offers more rapid changes in blood pressure typically over a wider range. This approach has been questioned on the basis of direct effects on the vessels (Kienbaum and Peters 2004). As we have discussed previously in detail, there are distinct advantages and disadvantages to both methods with the potential for confounding factors with either approach (Taylor et al. 2014). While the modified Oxford method is considered the gold standard approach for assessing cardiac BRS, it has some disadvantages for the assessment of sympathetic BRS. The process of quantifying sympathetic BRS relies upon the occurrence of MSNA bursts, which do not occur with every cardiac cycle. This severely limits the number of data points with which to plot a baroreflex slope. During the rise in pressure following the bolus injection of phenylephrine MSNA bursts can be inhibited altogether, which means that values of sympathetic BRS will often be determined mostly from the fall in pressure, following the sodium nitroprusside bolus (Dutoit et al. 2010). The use of spontaneous techniques in the current study allows these issues to be overcome as well as an opportunity to investigate the findings of Dutoit et al. (2010) using alternative approaches. Although the capacity of spontaneous baroreflex techniques to eliminate nonbaroreflex stimuli has been questioned, it is suggested that they hold predictive power (Diaz and Taylor 2006), thus providing useful information about baroreflex function as an alternative to methods where blood pressure changes are driven externally.

Interestingly, out of the six participants whose data were excluded due to a lack of cardiac BRS sequences, five had below-average values for sympathetic BRS_inc_ and all six had below-average values for sympathetic BRS_total_. The lack of cardiac baroreflex sequences itself may be interpreted as a sign of poor cardiac BRS and, consistent with the current findings, these individuals also exhibited low sympathetic BRS. Alternatively, the failure to obtain significant cardiac and sympathetic baroreflex slopes may be due to the existence of a nonlinear relationship between blood pressure and R-R interval or MSNA. While the example in Figure2 illustrates a significant relationship between diastolic pressure and MSNA, the bin representing the lowest diastolic pressure does not follow the linear trend, with MSNA bursts being much larger than those in the higher pressure bins. The process of removing data sets due to a lack of significant baroreflex slopes is common practice and, to the best of our knowledge, has not been questioned. Eliminating the results entirely from the investigation, based on insignificant linear regression slopes, could mean that useful information about blood pressure regulation in those individuals is ignored. Alternative methods for dealing with nonlinear relationships may be an important analytical problem worth investigating in baroreflex research. Previous studies indicate that MSNA burst incidence is closely related to diastolic BP, and is therefore more successful than MSNA burst area (Kienbaum et al. 2001; Hart et al. 2010). The total MSNA method for quantifying sympathetic BRS has been associated with both low (Hart et al. 2010) and high success rates (Keller et al. 2006). In the current study, the total MSNA method was only marginally less successful (39 successful baroreflex slopes) than the MSNA burst incidence method (42 successful baroreflex slopes). The sympathetic BRS _total_ method incorporates both MSNA burst amplitude, unlike the burst incidence method, and therefore it could be argued that it provides a better overall indication of sympathetic BRS than using MSNA burst incidence alone. Furthermore, Fairfax et al. (2013) recently demonstrated that MSNA burst amplitude has more influence than MSNA burst frequency on vascular conductance. In other words, clusters of bursts with higher amplitudes lead to greater reductions in blood vessel diameter than clusters of smaller but more numerous bursts (when total MSNA remains the same). Given its influence on the vasculature, it therefore seems logical to incorporate MSNA burst amplitude in the quantification of sympathetic BRS.

## Conclusions

In healthy, young females there is a correlation between cardiac and sympathetic baroreflex sensitivity. In this group, cardiac BRS may predict a small portion of the variance in baroreflex modulation of MSNA burst incidence and total MSNA. In contrast, this relationship appears not to be present in young males. We therefore conclude that cardiac BRS is not appropriate for estimating overall baroreflex function in healthy, young individuals. This relationship warrants further investigation, particularly in clinical and aging populations.

## Conflict of Interest

None declared.
